# Supplementary material for: Distributed acoustic sensing of microseismic sources and wave propagation in glaciated terrain
Source: Nat Commun. 2020 May 15;11:2436. doi: 10.1038/s41467-020-15824-6 (PMC7229220; doi:10.1038/s41467-020-15824-6)
Supplement: Supplementary file 1 — Supplementary Information [file 41467_2020_15824_MOESM1_ESM.pdf]

Supplementary Information for

Distributed Acoustic Sensing of Microseismic Sources and Wave Propagation in Glaciated Terrain

by F. Walter, D. Gräff, F. Lindner, P. Paitz, M. Köpfli, M. Chmiel, and A. Fichtner

Supplementary Figures

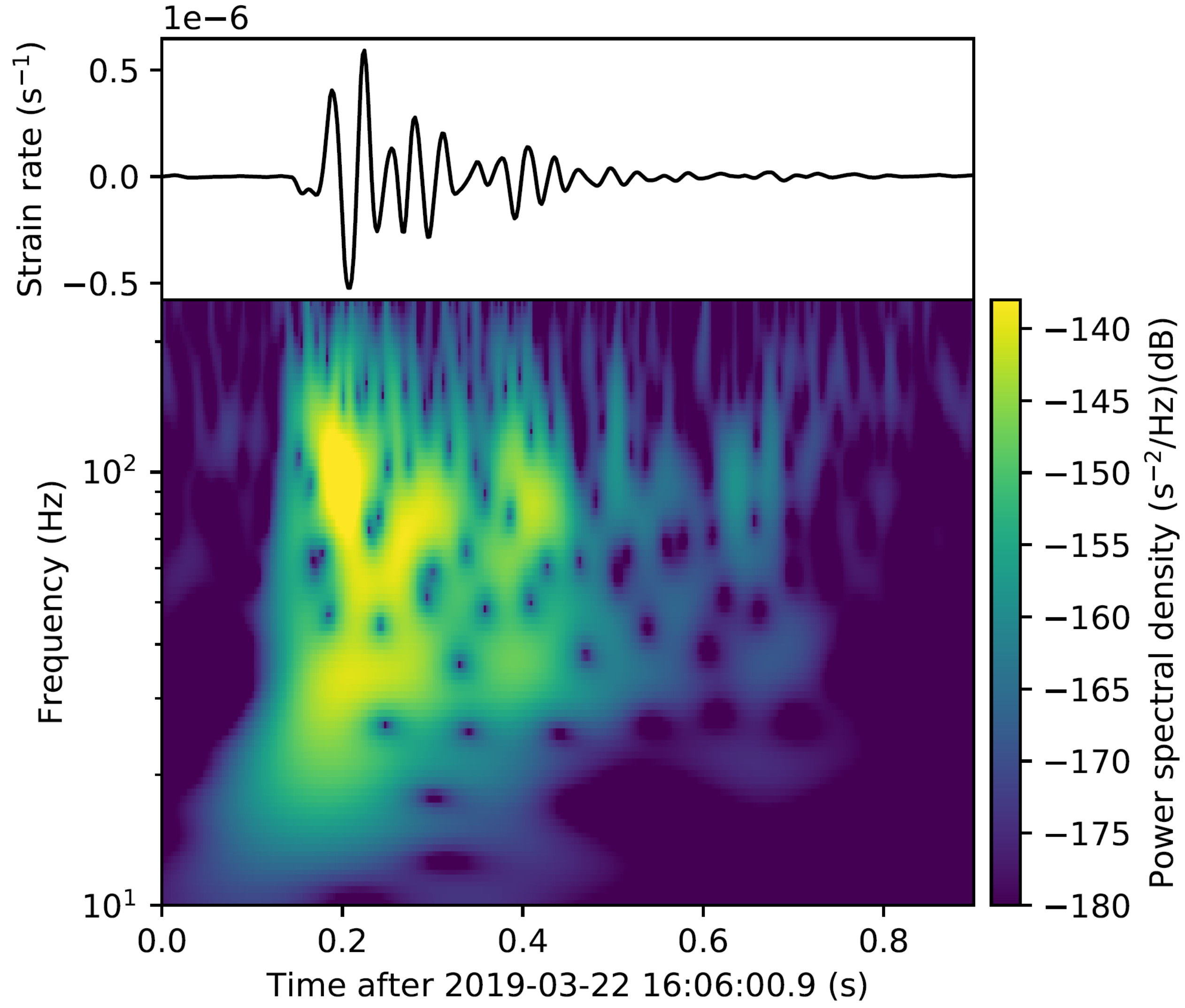

Supplementary Figure 1: Surface Icequake Spectrogram. Spectrogram of surface icequake shown in Figure 2 recorded on the southern corner of the DAS cable. Time series was filtered as in Figure 2.

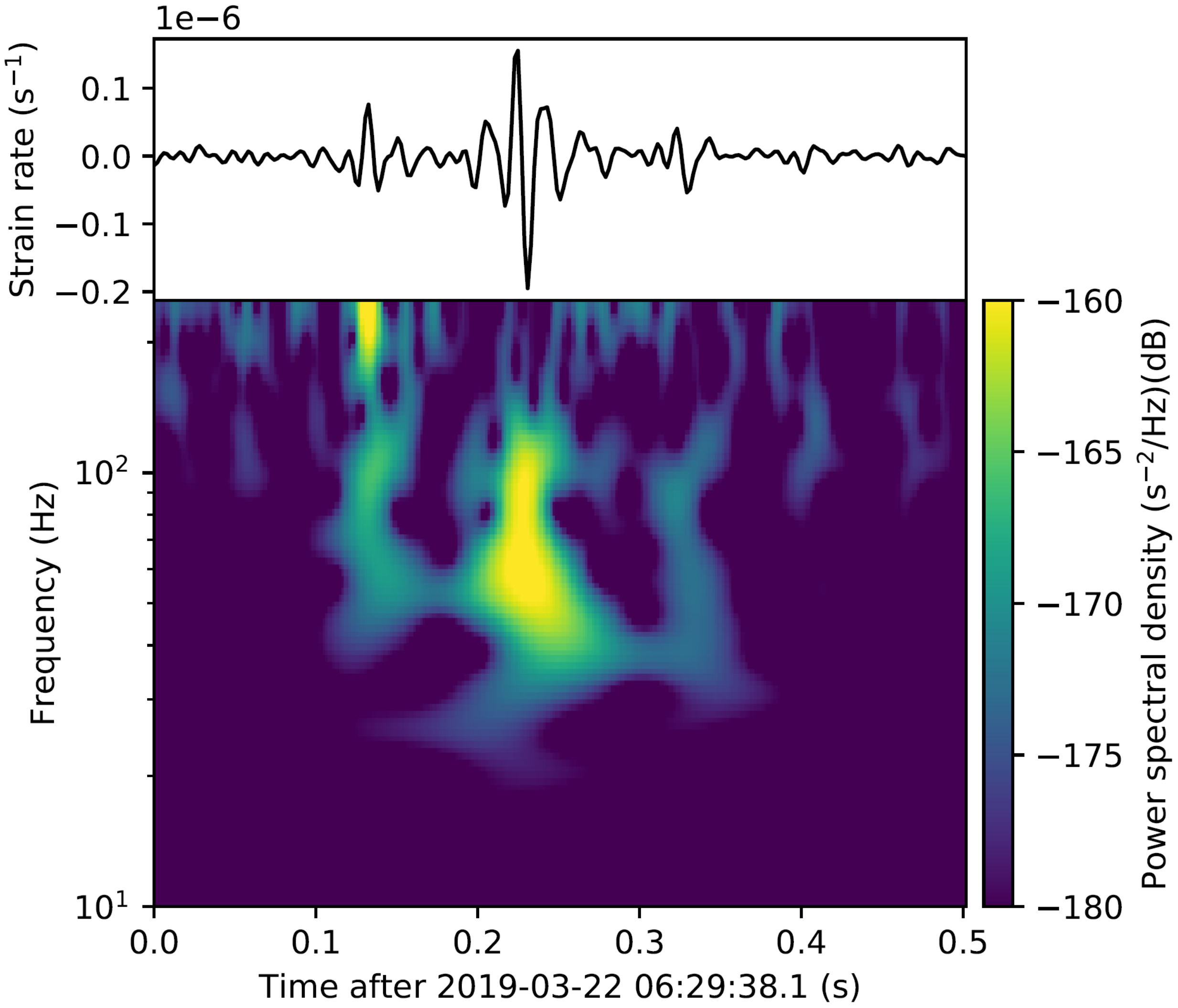

Supplementary Figure 2: Stick-Slip Icequake Spectrogram. Spectrogram of stick-slip icequake shown in Figures 2 and 3 recorded on the southern corner of the DAS cable. Time series was filtered as in Figure 2.

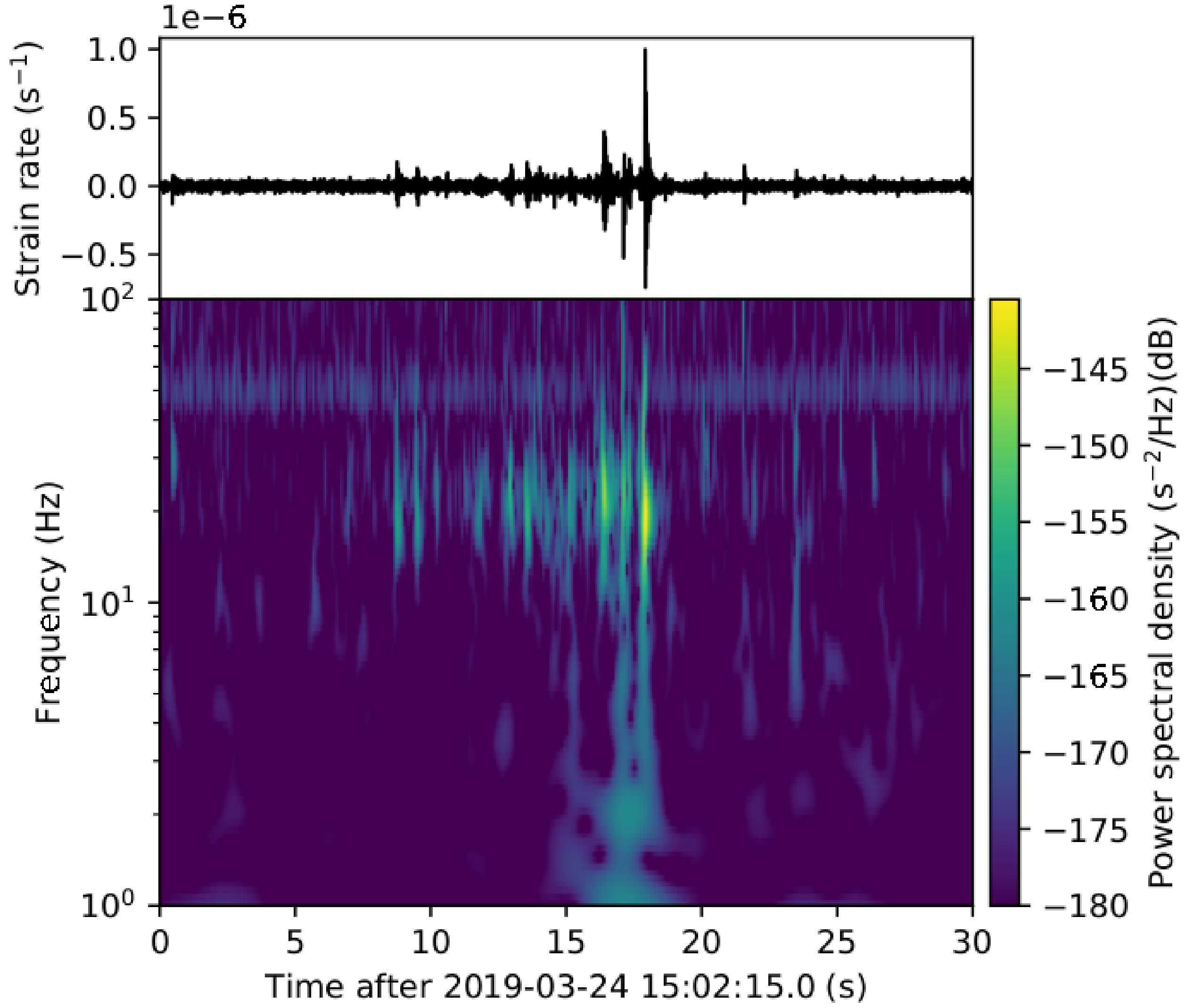

Supplementary Figure 3: Rockfall Spectrogram. Spectrogram of the rockfall shown in Figures 2 and 5 recorded on the southern corner of the DAS cable. The impulses correspond to impacts of individual blocks on the mountain slope or glacier. The signal energy resides mostly below a few tens of Hz, typical for surface waves from distant sources. Note the different spectrogram y-scale compared to Figures S1 and S2. Time series was filtered as in Figure 2.

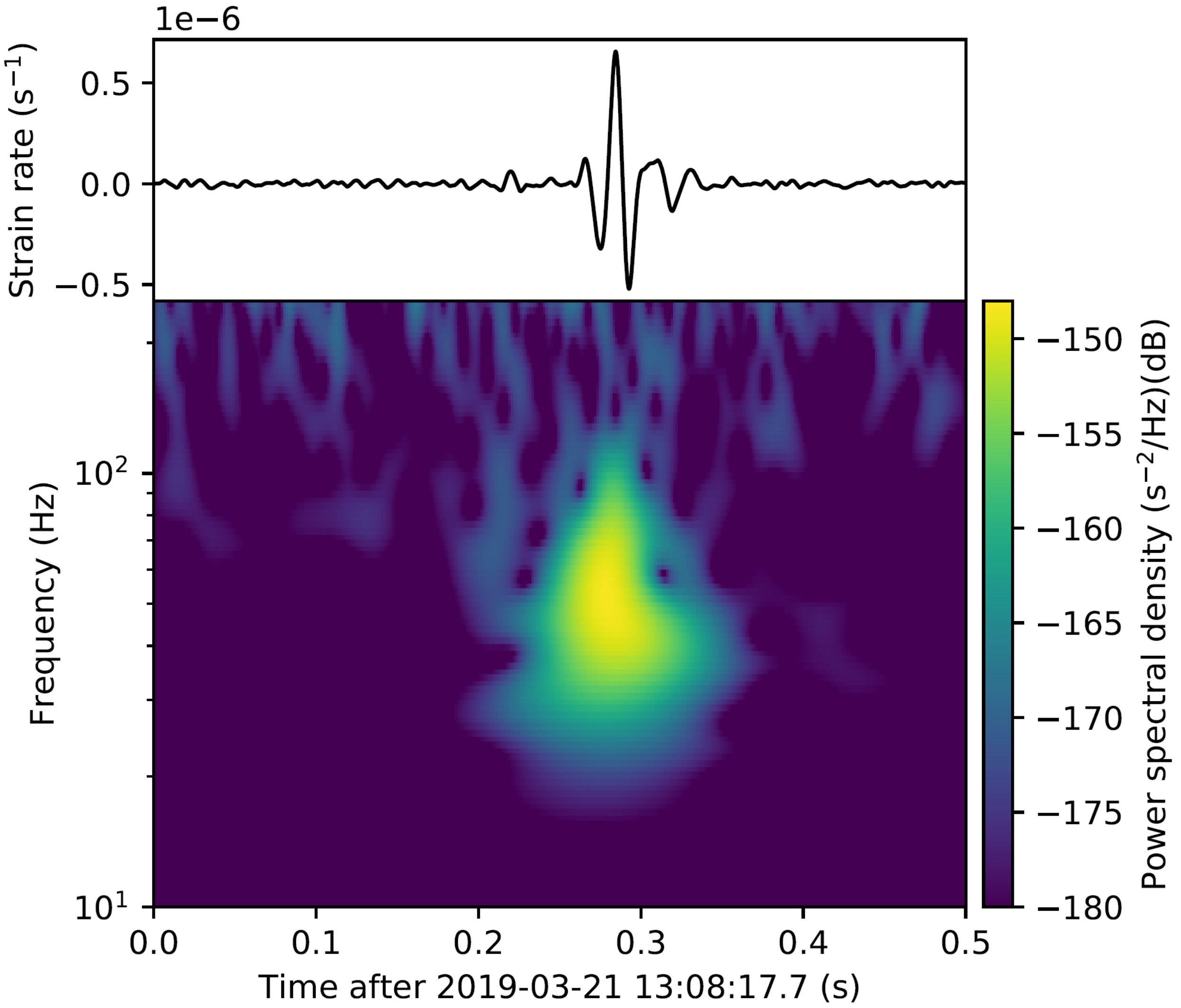

Supplementary Figure 4: Explosion Spectrogram. Spectrogram of explosion shown in Figure 2 recorded on the northwestern corner of the DAS cable. Note different spectrogram y-scale compared to Figures S1-S3. Time series was filtered as in Figure 2.

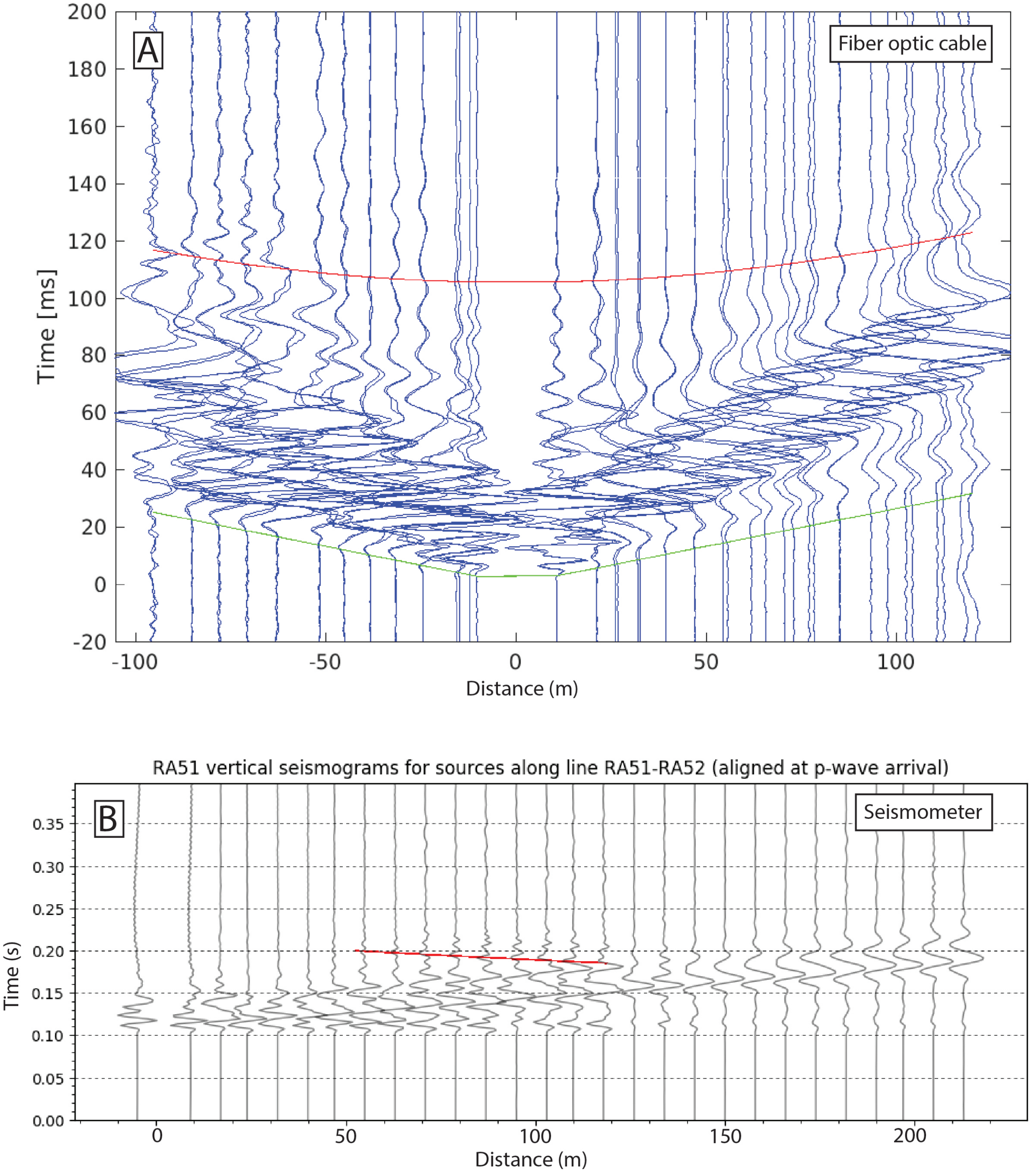

Supplementary Figure 5: Explosion Shot Gathers. Along the northern cable section a total of 28 explosive charges (125 g of Riodin HE) were drilled through the snow and set off ca. 30 cm within the ice to investigate the performance of the DAS system in active seismic experiments. (**A**) Explosion seismograms of all channels along this segment are shown for a single shot. Green and red curves indicate the expected arrival times of direct and reflected P-waves traveling at 3800 m/s and the dominant phase is the Rayleigh wave. The lack of a clear reflection is likely related to the poor ice-cable coupling resulting from the damping snow layer. (**B**) Shot seismograms recorded on Station RA51 with x-axis indicating source-station distances and y-axis indicating time relative to direct P-wave arrival. Each time series represents a single shot and all shots are located along the northern

cable section connecting RA51 with RA52. In contrast to the shot gather of Panel A, a small secondary arrival is visible (red line) in Panel B, which we interpret as the basal P reflection.

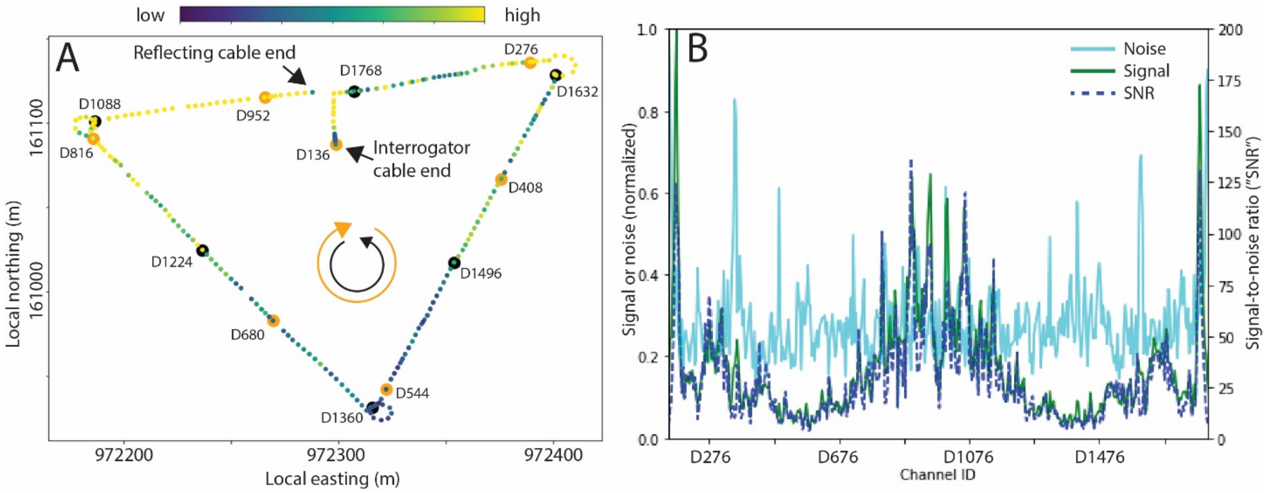

Supplementary Figure 6: Spatial distribution of noise and signal strengths. Stick-slip event shown in Figures 2-4 and S2 are analyzed. (**A**) Cable layout with sensing channels color coded by ratio of signal strength to noise strength. Signal strength is defined as maximum within 0.3 s time window including main arrivals and noise strength is defined as standard deviation of a 0.4 second pre-event time window. Absolute values of color code range correspond to Panel B (right y-axis). Some channels names along the cable are labeled with large orange markers denoting the distances (in meters) from interrogator to reflecting cable end. Large black markers correspond to channels after reflection. (**B**) Signal and noise strengths plotted against channel ID's.

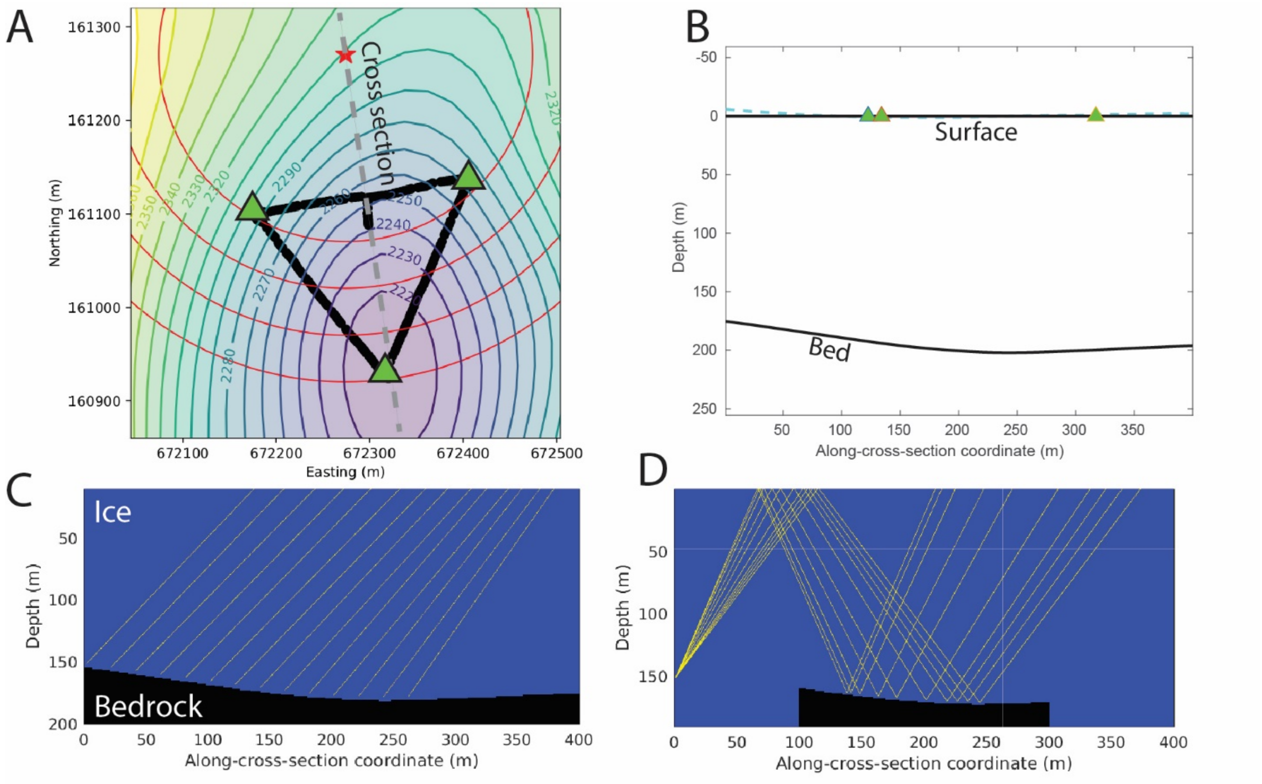

Supplementary Figure 7: 2D ray tracing. (**A**) Color-coded glacier bed topography (contour lines in meters), seismometers (green triangles), fiber optic cable (black triangular outline), stick-slip event hypocenter (red star), longitudinal cross sectional path (dashed grey line) and red circles around event epicenter with radii from 200 - 350 m in 50 m steps. The red circles around the epicenter show how the cable segments are projected onto the modelled cross section where the bedrock elevation is much lower than below their actual location (e.g. the bedrock elevation below the upper left corner of the cable triangle is at ~2295 m and it is projected to the cross sectional part where it is at only 2235 m). (**B**) Longitudinal cross section after 30-point smoothing and counterclockwise coordinate rotation to obtain a horizontal surface (cyan dashed line shows the actual surface topography and black line the flat approximation). (**C**) Ray tracing of critically refracted waves traveling along the ice-bed interface. (**D**) Doubly reflected rays. For illustration purposes the reflection region is drawn separately in a homogeneous ice halfspace, because the bed reflection region was lifted by 3 m with respect to the bed shown in Panel C.

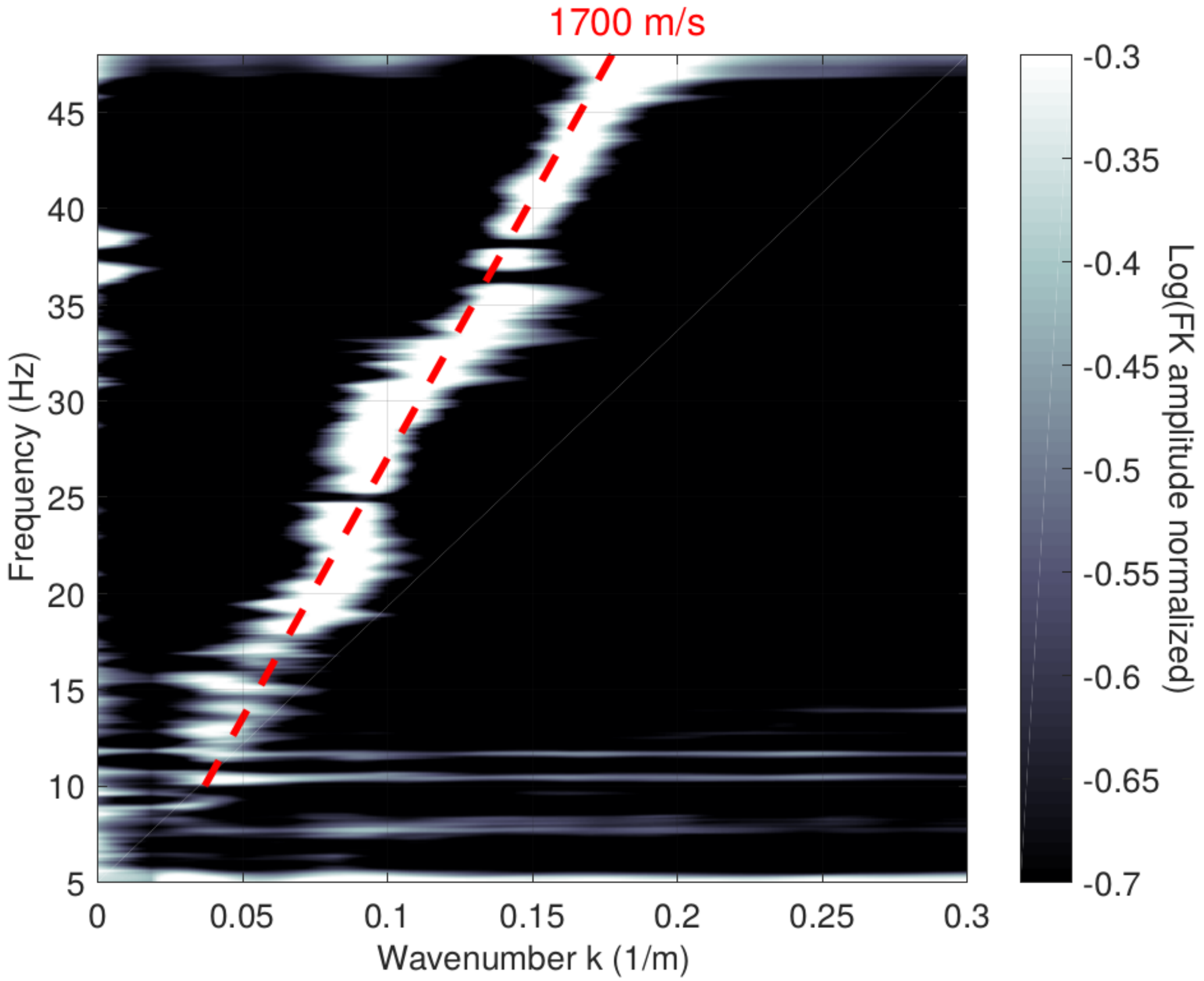

Supplementary Figure 8: F-K Transform of Noise Correlations. 2D Fourier Transform of a one-day DAS record from 24 March 2019 using the eastern triangle segment, only. For wavenumbers above 0.04 m^{-1} (wavelength below 160 m) and frequencies above 10 Hz, the frequency-wavenumber peaks lie near a straight line whose slope describes the propagation velocity of a Rayleigh wave. In contrast, at lower wavenumbers, this linear relation between frequency and wavenumber is no longer apparent which we suggest is a manifestation of the 220 m length limit of the cable segment.

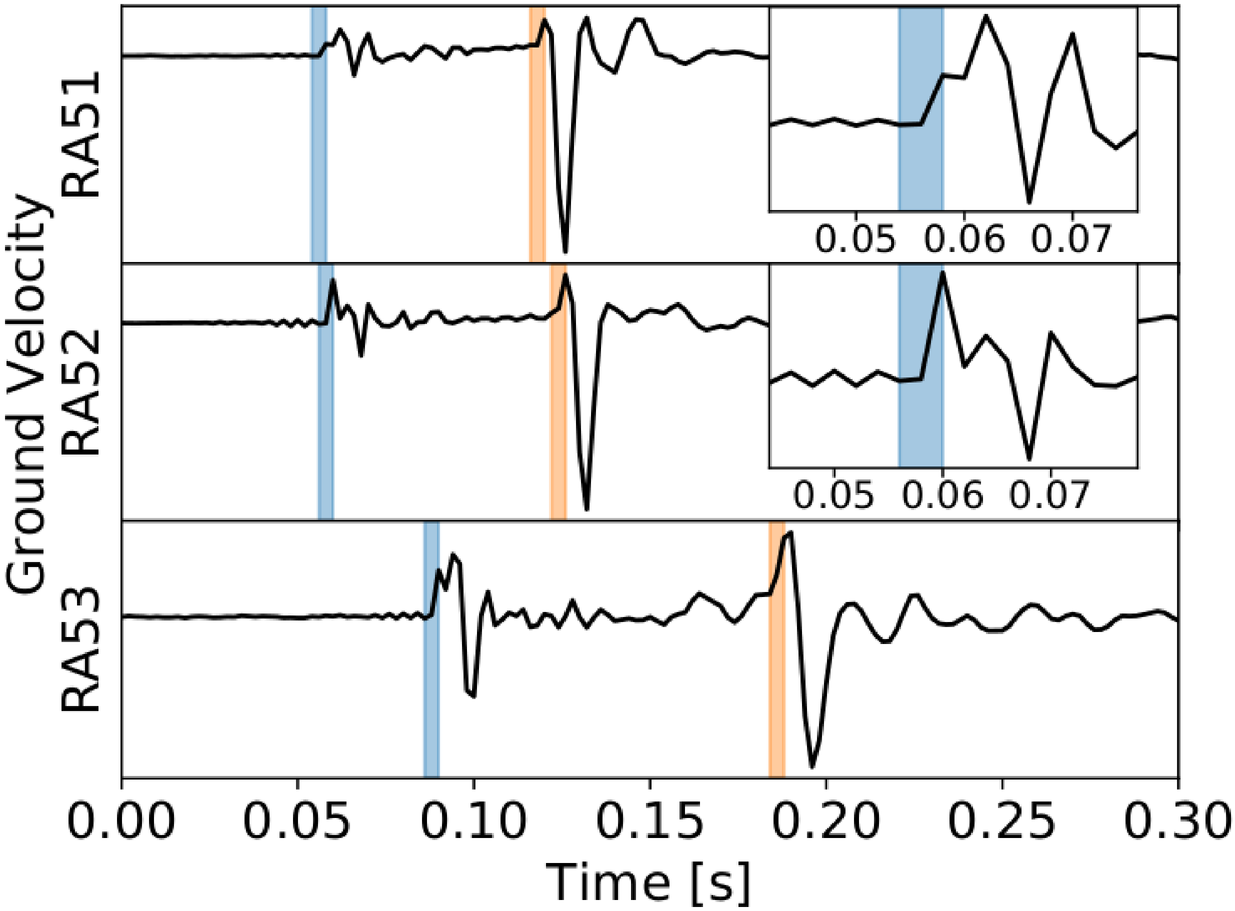

Supplementary Figure 9: Arrival Time Picking. Picking uncertainties of P (blue) and S-waves (orange) on stick-slip records of on-ice borehole stations RA51-53 drilled ca. 3 m into the ice. Notice that at all stations, the first P-wave break is suddenly interrupted by a downward motion after approximately 1 sample (2 ms). This is the results of a "ghost", i.e. reflection off the ice surface, which reaches the borehole seismometer slightly later than the direct wave. Prior to the first P-wave break, acausal precursory oscillations at the Nyquist frequency of 250 Hz are visible, which may mask weaker refracted arrivals.

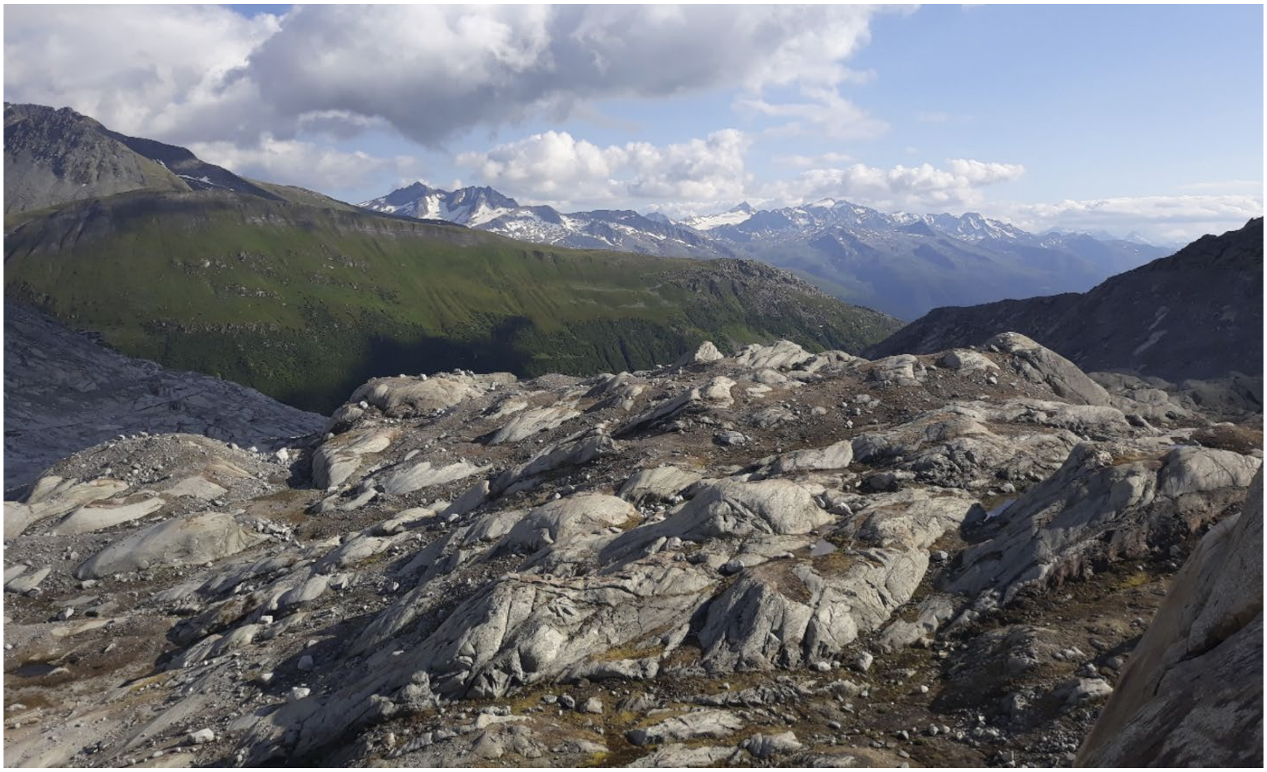

Supplementary Figure 10: Glacier Forefield. The retreat of Rhonegletscher's tongue exposes bedrock. Photograph by Dominik Gräff.

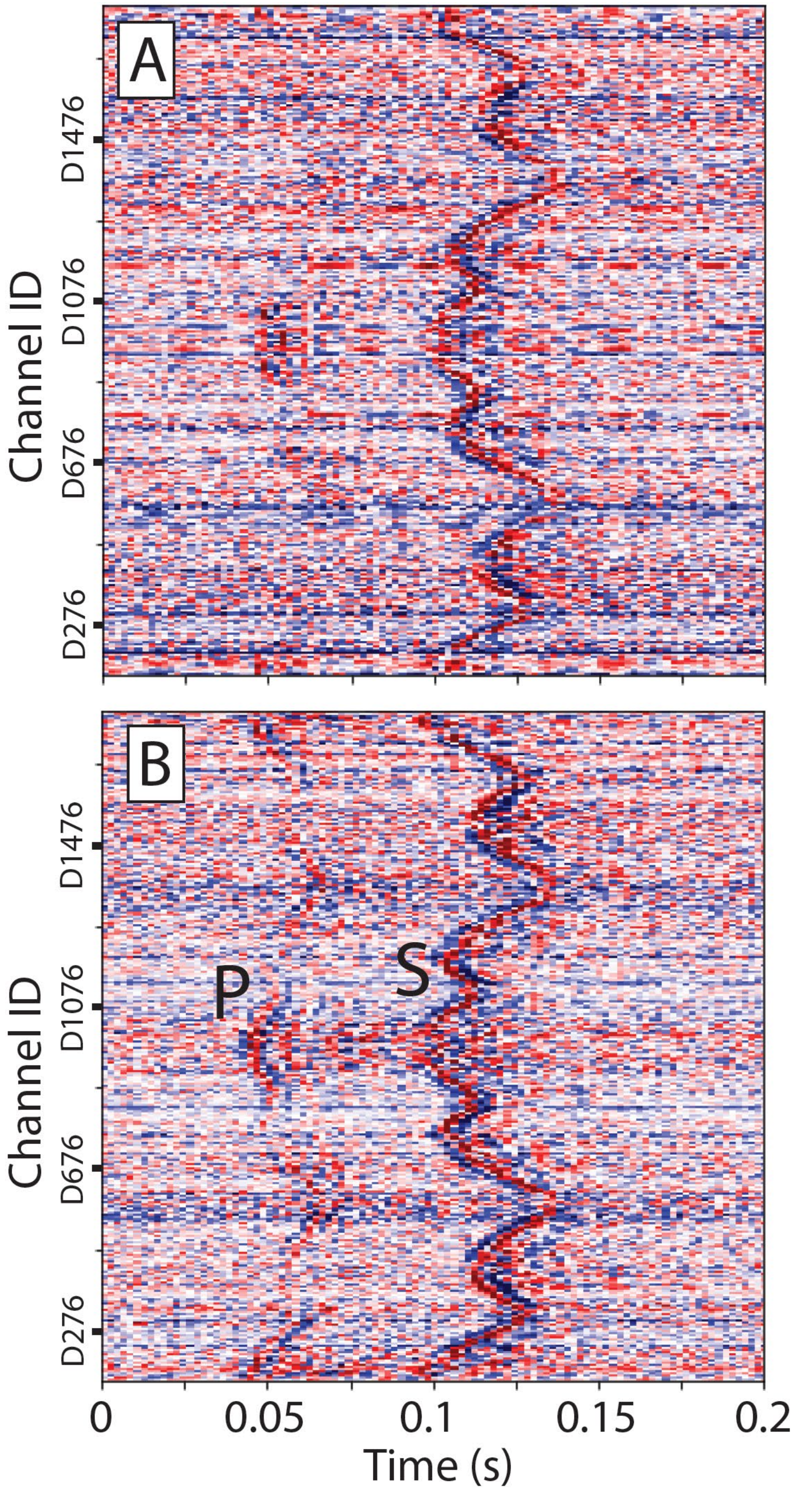

Supplementary Figure 11: Signal stacking of stick-slip repeaters. (**A**) Single stick-slip record of DAS system (cluster epicenter shown in Figure 4B). (**B**) Stack of 5 events with visibly improved signal-to-noise ratio. P-waves, in particular are better visible in the stack.

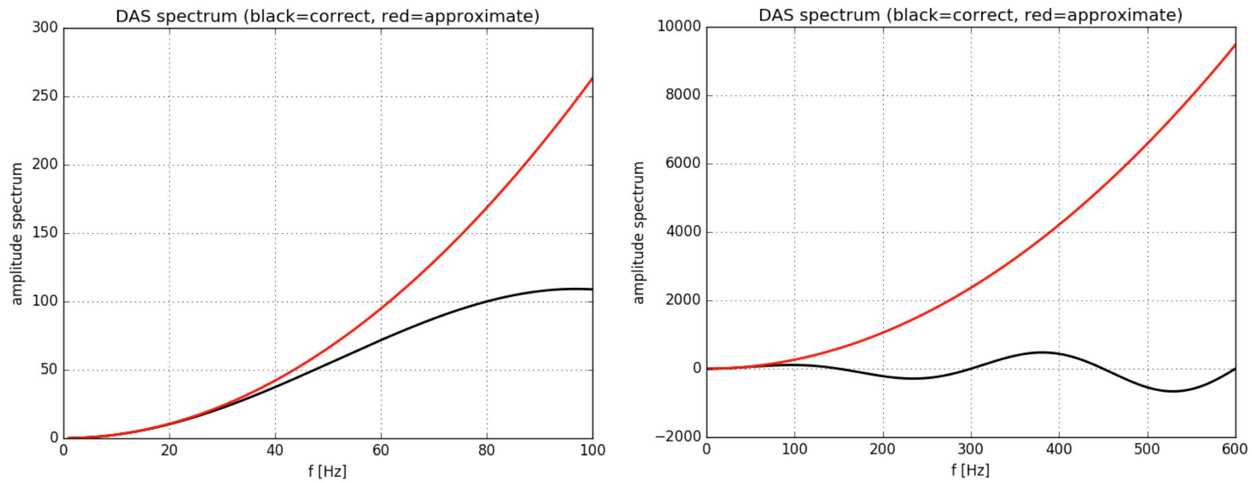

Supplementary Figure 12: DAS Amplitude Spectra. Correct (black, Equation (11) in the supplementary notes) are compared to approximated (red, Equation (14) in the supplementary notes) amplitude spectra of the DAS recordings. The figure to the left is a zoom into the figure to the right, roughly showing the frequency range where the approximation is valid. Here, this is up to around 50 Hz. Thus, up to that frequency, DAS recordings can be considered as scaled acceleration recordings.

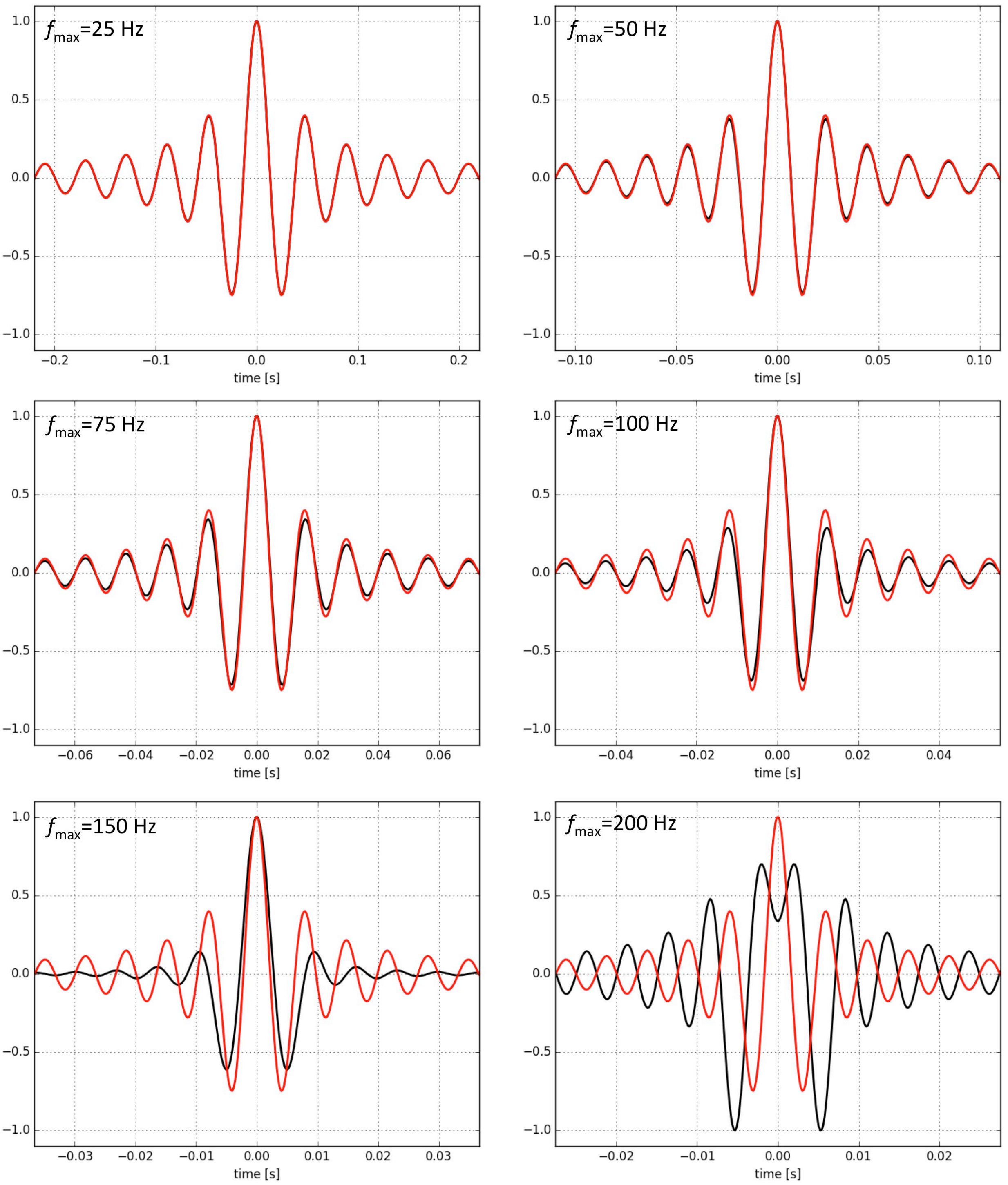

Supplementary Figure 13: Pulse Shape Comparison. DAS (black) are compared to acceleration (red) pulses shapes as a function of the maximum frequency f_{max}. The figure confirms the observation from Supplementary Figure 12 that the DAS and acceleration pulse shapes are practically identical up to frequencies of 50 Hz. The pulse shapes start to differ substantially only for frequencies above 100 Hz, which corresponds to a wavelength of 15 m, i.e., 1.5 times the gauge length.

Supplementary Notes

In these supplementary notes, we derive the phase and amplitude of plane waves recorded by DAS. This has implication for our ability to measure traveltimes in DAS waveforms, depending on the frequency range that we consider. The major conclusion is that DAS recordings of strain rate are practically identical to a scaled acceleration recording for wavelengths exceeding the gauge length. Only for wavelengths shorter than about half the gauge length, pulse shapes start to differ more substantially, potentially leading to biased traveltime measurements.

Supplementary Note 1: Plane waves recorded by DAS

We consider a plane wave displacement at position $\mathbf{x}$ in the frequency domain,

$$\mathbf{u}(\mathbf{x}, \omega) = \mathbf{A} e^{-i(\mathbf{k}^T\mathbf{x} + k\Delta)}\,, \tag{1}$$

where $\mathbf{A}$ is the polarisation vector, $\mathbf{k}$ is the wave number vector of length $|\mathbf{k}| = k$, and ω is the circular frequency. In the time domain, Equation (1) corresponds to a plane δ-pulse arriving at the observation position $\mathbf{x} = \mathbf{0}$ at time $t = \Delta/c$, where c is the phase velocity of the assumed homogeneous medium. Wave number vector and circular frequency are related by the phase velocity $c = \omega/k$.

DAS measures strain rate averaged over a certain length, the gauge length. So, we first compute the strain tensor related to the displacement in (1):

$$\epsilon_{ij} = \frac{1}{2}\left(\frac{\partial u_i}{\partial x_j} + \frac{\partial u_j}{\partial x_i}\right) = -\frac{i}{2}(A_i k_j + A_j k_i)\, e^{-i(\mathbf{k}^T\mathbf{x} + k\Delta)}\,. \tag{2}$$

The optical fibre is oriented in a certain direction, specified by the unit vector $\mathbf{e}$. The strain in that direction is

$$\epsilon = e_i \epsilon_{ij} e_j = -i\,(\mathbf{e}^T\mathbf{A})(\mathbf{e}^T\mathbf{k})\, e^{-i(\mathbf{k}^T\mathbf{x} + k\Delta)}\,. \tag{3}$$

More generally, the orientation is position-dependent, i.e., $\mathbf{e} = \mathbf{e}(\mathbf{x})$. Here, for simplicity, we consider $\mathbf{e}$ constant. In the next step, we average the strain ϵ over a certain distance along the fibre. For this, we parameterise the position $\mathbf{x}$ as

$$\mathbf{x}(\tau) = \tau\mathbf{e}\,, \qquad \text{with} \quad \tau \in (-\ell/2, \ell/2)\,. \tag{4}$$

This means, we consider a gauge length ℓ, half of which is left of the measurement location $\mathbf{x} = \mathbf{0}$, and the other half is right of it. Carrying out the averaging integral, we find

$$\bar{\epsilon} = \frac{1}{\ell}\int\limits_{\tau=-\ell/2}^{\ell/2} \epsilon(\tau)\, d\tau = \frac{1}{\ell}(\mathbf{e}^T\mathbf{A}) e^{-i(\mathbf{k}^T\mathbf{e}\tau + k\Delta)}\Big|_{-\ell/2}^{\ell/2} = -2\frac{i}{\ell}\,(\mathbf{e}^T\mathbf{A})\sin\left(\frac{\ell}{2}\mathbf{k}^T\mathbf{e}\right) e^{-ik\Delta}\,. \tag{5}$$

Taking the time derivative of (5) in the frequency domain (multiplication by $i\omega$), we finally obtain the stain rate averaged over the gauge length:

$$\dot{\bar{\epsilon}} = \frac{2kc}{\ell}\,(\mathbf{e}^T\mathbf{A})\sin\left(\frac{\ell}{2}\mathbf{k}^T\mathbf{e}\right) e^{-ik\Delta}\,. \tag{6}$$

Equation (6) holds for both P and S waves, as we have not specified the polarisation direction.

Supplmentary Note 2: Amplitude spectrum

It is clear from Equation (6) that the measurement of strain rate and the act of averaging over the gauge length ℓ has modified the amplitude spectrum from the constant in the displacement Equation (1) to

$$\frac{2kc}{\ell}\,(\mathbf{e}^T\mathbf{A})\sin\left(\frac{\ell}{2}\mathbf{k}^T\mathbf{e}\right) = \frac{2kc}{\ell}\,(\mathbf{e}^T\mathbf{A})\sin\left(\pi\frac{\ell}{\lambda_a}\right)\,, \tag{7}$$

where

$$\lambda_a = \frac{\lambda}{\mathbf{n}^T\mathbf{e}}\,, \tag{8}$$

is the effective wave length, and $\mathbf{n}$ is the unit-length propagation direction parallel to $\mathbf{k}$. (This is the wavelength seen by the fibre. When the propagation direction is exactly along the fibre, we have $\mathbf{n} \parallel \mathbf{e}$ and $\lambda_a = \lambda$. In the other extreme case we have $\mathbf{n} \perp \mathbf{e}$, and the apparent wavelength is infinite.)

The averaging over the gauge length ℓ acts to eliminate certain frequencies from the spectrum. In fact, the spectral amplitude vanishes for all frequencies ω for which an integer multiple of λ_a fits into the gauge length, that is,

$$\ell = n\lambda_a\,, \quad n \in \mathbb{N}\,. \tag{9}$$

Interestingly, Equation (11) also implies that the contributions of frequencies with

$$\ldots\,, \quad \frac{\ell}{4} < \lambda_a < \frac{\ell}{3}\,, \quad \frac{\ell}{2} < \lambda_a < \ell\,, \tag{10}$$

are added to the complete time-domain DAS recording with reversed amplitude. Obviously, this is only something that happens at high frequencies.

Supplementary Note 3: Phase spectrum and traveltimes

Since the amplitde spectrum is obviously complicated, we consider the special case of low frequencies. For this, we express (6) in terms of ω:

$$\dot{\epsilon} = 2\frac{\omega}{\ell}\,(\mathbf{e}^T\mathbf{A})\sin\left(\frac{\omega\ell}{2c_a}\right)e^{-ik\Delta}\,, \tag{11}$$

where we introduced the apparent velocity

$$c_a = \frac{c}{\mathbf{n}^T\mathbf{e}}\,. \tag{12}$$

For frequencies that are low in the sense of

$$|\omega| < \frac{\pi c_a}{\ell}\,, \quad \text{or} \quad \ell < 2\lambda_a \tag{13}$$

we may approximate the $\sin$ in (11):

$$\dot{\epsilon} = \frac{\omega^2}{c_a}(\mathbf{e}^T\mathbf{A})e^{-ik\Delta}\,. \tag{14}$$

Equation (14) shows that the spectrum of the DAS recording is identical to the spectum of displacement acceleration (i.e., proportional to ω^2), just scaled by the inverse apparent velocity c_a and the orientation term $\mathbf{e}^T\mathbf{A}$. In this long-wavelength scenario, the DAS measurement will look like a displacement acceleration recording at $\mathbf{x} = \mathbf{0}$.

In any case, we see that the phase of the DAS response (6) is exactly the same as the phase of the displacement (1) measured at $\mathbf{x} = \mathbf{0}$, namely $e^{-ik\Delta}$. Thus, the whole prodecure of computing

strain, averaging over the gauge length, and taking a time derivative has not changed the phase. As a consequence, we still observe a pulse centred at an arrival time of $t = \Delta/c$. What has changed is the pulse shape in the time domain. However, since the frequency-domain amplitude (7) is real-valued, it is still symmetric around the arrival time, meaning that we do not expect any picking error due to a skewed pulse shape.

Supplementary Note 4: Examples

To illustrate the above developments, we consider a specific case where the gauge length is ℓ =10 m, and the apparent velocity is c_a =1700 m/s. Supplementary Figure 12 shows the amplitude spectrum of a DAS recording compared to the aplitude spectrum of an acceleration recording. The latter also corresponds to the low-frequency approximation of the DAS response. Supplementary Figure 13 contains a collection of pulse shapes as a function of frequency. The main conclusion is that the DAS recording is practically identical to a scaled acceleration recording for frequencies up to around 50 Hz. Only above 100 Hz the pulse shapes start to differ more substantially. Still, up to around 150 Hz, the traveltime picking error (based on picking the maximum) is exactly zero.
